# Alzheimer's disease‐like neuropathology in three species of oceanic dolphin

**DOI:** 10.1111/ejn.15900

**Published:** 2022-12-27

**Authors:** Marissa C. Vacher, Claire S. Durrant, Jamie Rose, Ailsa J. Hall, Tara L. Spires‐Jones, Frank Gunn‐Moore, Mark P. Dagleish

**Affiliations:** ^1^ Institute of Biology Leiden University Leiden the Netherlands; ^2^ Centre for Discovery Brain Sciences and UK Dementia Research Institute University of Edinburgh Edinburgh UK; ^3^ Sea Mammal Research Unit, Scottish Oceans Institute University of St. Andrews Fife UK; ^4^ School of Biology University of St Andrews Fife UK; ^5^ School of Biodiversity, One Health and Veterinary Medicine, Pathology Department University of Glasgow Scotland UK

**Keywords:** amyloid plaques, beta amyloid, cetacean, immunohistochemistry, neurofibrillary tangles, odontocetes, tau

## Abstract

Alzheimer's disease (AD) is the most common neurodegenerative disease and the primary cause of disability and dependency among elderly humans worldwide. AD is thought to be a disease unique to humans although several other animals develop some aspects of AD‐like pathology. Odontocetes (toothed whales) share traits with humans that suggest they may be susceptible to AD. The brains of 22 stranded odontocetes of five different species were examined using immunohistochemistry to investigate the presence or absence of neuropathological hallmarks of AD: amyloid‐beta plaques, phospho‐tau accumulation and gliosis. Immunohistochemistry revealed that all aged animals accumulated amyloid plaque pathology. In three animals of three different species of odontocete, there was co‐occurrence of amyloid‐beta plaques, intraneuronal accumulation of hyperphosphorylated tau, neuropil threads and neuritic plaques. One animal showed well‐developed neuropil threads, phospho‐tau accumulation and neuritic plaques, but no amyloid plaques. Microglia and astrocytes were present as expected in all brain samples examined, but we observed differences in cell morphology and numbers between individual animals. The simultaneous occurrence of amyloid‐beta plaques and hyperphosphorylated tau pathology in the brains of odontocetes shows that these three species develop AD‐like neuropathology spontaneously. The significance of this pathology with respect to the health and, ultimately, death of the animals remains to be determined. However, it may contribute to the cause(s) of unexplained live‐stranding in some odontocete species and supports the ‘sick‐leader’ theory whereby healthy conspecifics in a pod mass strand due to high social cohesion.

## Graphical abstract

Vacher et al used immunohistochemistry to look for Alzheimer's‐like pathology in the brains of aged long‐finned pilot whales, white‐beaked dolphins, Risso's dolphins, harbour porpoises and a bottle‐nosed dolphin that were found beached on the shores of Scotland. All aged animals had amyloid plaque pathology, and three species exhibited both plaques and accumulation of phosphorylated tau in neurons. The simultaneous occurrence of amyloid‐beta plaques and hyperphosphorylated tau in the brains of odontocetes shows that these three species develop Alzheimer's‐like neuropathology spontaneously.

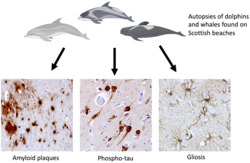

**Abbreviations**
AD: Alzheimer's disease
APs: amyloid plaques
Aβ: amyloid‐beta peptide
GFAP: glial fibrillary acidic protein
Iba1: ionized calcium binding adaptor molecule 1
NFTs: neurofibrillary tangles
NPs: neuritic plaques
NTs: neuropil threads
PRLS: post‐reproductive lifespan
pTau: hyperphosphorylated tau protein

## 1 INTRODUCTION

Alzheimer's disease (AD) is a neurodegenerative disorder and one of the primary causes of disability and dependency affecting approximately 30 million elderly people worldwide (Alzheimer's Association, 2015). Its economic impact on the United Kingdom alone has been estimated to be over £20 billion, annually (Prince et al., 2014). Although clinical disease and the severity of neuropathology are not necessarily correlated linearly (Nelson et al., 2009), neurodegeneration and disease progression are typical once the pathognomonic lesions are present beyond a certain stage, gradually impairing memory, learning, communication skills and the ability to perform daily activities (Mufson et al., 2008; Perl, 2021; Whitehouse et al., 2004). Alois Alzheimer first described the characteristic amyloid plaques (APs) and neurofibrillary tangles (NFTs) over 100 years ago (Cipriani et al., 2011). Despite continuous research since discovery, there is still no preventive or curative treatment for AD, nor any treatment to prevent its progressive course.

The accumulation of amyloid‐beta peptide (Aβ), eventually forming APs, and the NFTs, composed of paired helical filaments of hyperphosphorylated tau protein (pTau), have been studied extensively in humans and animal models. Studies in the brains of human patients remain the most critical route for characterizing and understanding the condition as AD appears to be exclusive to humans (Walker & Jucker, 2017; Youssef et al., 2016), and the absence of an accurate, valid experimental animal model, spontaneous or induced and that completely captures the human phenotype, has hindered research on the etiology and pathogenesis of the disease. Furthermore, studies using these incomplete (including transgenic) animal models have yielded contradicting results thereby impeding progress in developing potential treatments (Orta‐Salazar et al., 2016).

Two recent reports in cetaceans, one suggesting deep diving beaked whales are more susceptible to AD‐like pathology due to the hypoxia associated with their foraging activities (Sacchini et al., 2020) and a report of a single captive aged (40‐year‐old) bottlenose dolphin (Stylianaki et al., 2019), and one reporting the presence of Aβ and tau‐related pathology in the brains of aged pinnipeds (Takaichi et al., 2021) suggest these species require in‐depth investigation. Furthermore, neuropil threads (NTs) have been reported in the brains of four striped dolphins (*Stenella coeruleoalba*) and one bottlenose dolphin (*Tursiops truncatus*), and small APs were found in three striped dolphins (Gunn‐Moore et al., 2018). As the amino acid sequence of Aβ in the Risso's dolphin (*Grampus griseus*), striped dolphin and bottlenose dolphin, is identical to that of humans, it was proposed that some species of cetaceans (whales and dolphins), specifically odontocetes (toothed cetaceans), might develop AD‐like pathology spontaneously (Gunn‐Moore et al., 2018).

The view that the presence of Aβ and pTau are purely disadvantageous is being re‐evaluated as evidence that AD is an antagonistic pleiotropic effect is increasing, and this suggests the disease is a result of genes that control both beneficial and detrimental traits (Austad & Hoffman, 2018; Bufill et al., 2013; Hashimoto et al., 2018; Kent et al., 2020). Therefore, Aβ should not be regarded solely as a pathogenic factor as the peptide has various important physiological roles in the modulation of synaptic activity and neuronal survival (Kent et al., 2020; Parihar & Brewer, 2010; Pearson & Peers, 2006). Similarly, under normal physiological conditions, the equilibrium between phosphorylation and dephosphorylation of tau protein, controlled by various kinases and phosphatases, is a critical post‐translational modification that regulates cytoplasmic microtubules allowing growth and remodelling (Stoothoff & Johnson, 2005). These proteins are constitutive and should only be regarded as detrimental when there is an imbalance in their production and/or degradation along with other influencing factors (Ittner et al., 2017; Kent et al., 2020; Parihar & Brewer, 2010; Pearson & Peers, 2006; Stoothoff & Johnson, 2005).

The existence of menopause and a post‐reproductive lifespan (PRLS) in mammals is rare, and humans are the only terrestrial mammal known to have a significant PRLS (Ellis, Franks, Nattrass, Cant, et al., 2018). Despite this, PRLS has been reported in killer whales (*Orcinus orca*), short‐finned pilot whales (*Globicephala macrorhynchus*), belugas (*Delphinapterus leucas*), narwhals (*Monodon monoceros*) and false killer whales (*Pseudorca crassidens*) (Croft et al., 2017; Currie, et al., 2018; Ellis, Franks, Nattrass, Photopoulou et al., 2017; Foote, 2008; Marsh & Kasuya, 1968; Olesiuk et al., 1990), and its occurrence in these and, potentially, other odontocete cetaceans may make these animals susceptible to late‐onset diseases that have a genetic component, including AD. Most wild, free‐ranging animals with cognitive deficiencies are unlikely to survive if they are solitary, and any AD‐related pathology present would not be expected to exceed the stage associated with very early clinical disease. However, many cetacean species live in highly cooperative groups, especially odontocetes. Epimeletic (care‐giving) behaviour towards diseased or dying individuals has been recorded frequently in free‐ranging odontocetes of several species (Bearzi et al., 2017; Cockcroft & Sauer, 1990; Kuczaj et al., 2015). If individuals of these species develop clinical disease or impaired cognition, group members can aid their survival thereby allowing the severity of the pathology to progress.

Although epimeletic behaviour in animals is not unique to odontocetes (De Waal & Preston, 2017), it is exceptional in combination with PRLS and so odontocetes are, theoretically, likely to develop more advanced stages of aging‐associated disorders than other wild mammals.

## 2 MATERIALS AND METHODS

### 2.1 Aims and study design

Samples of specific brain regions from several species of free‐ranging marine odontocetes (that stranded, died and were examined as part of a long‐term stranding scheme) present in a national archive were examined by immunohistochemistry (IHC)/fluorescence for the presence of known markers of AD‐like neuropathology.

### 2.2 Cetacean brain tissue and case selection

The Scottish Government, specifically Marine Scotland, funds post‐mortem examinations, via the Scottish Marine Animal Stranding Scheme (SMASS), of cetaceans, pinnipeds and marine turtles that strand and die in Scottish coastal waters. For carcasses in a suitable state of preservation and within logistical limits, this includes removal of the brain, whole, and samples of a wide range of other tissues that are fixed in 10% neutral buffered formal saline prior to detailed histological examination. Routinely, coronal slices of fixed whole brain were made through the limbic and anterior paralimbic lobes of the cerebral cortex and the underlying basal ganglia, the supralimbic/paralimbic/limbic lobes and the underlying thalamus and the lingual lobe and underlying midbrain. Coronal slices were also made through the cerebellum and underlying pons, three levels of the medulla and a sagittal slice through the cerebellar vermis. Representative samples from these selected areas were trimmed, placed into large cassettes, processed through graded alcohols and embedded in paraffin wax prior to sectioning (4 μm) and mounting on glass microscope slides (HISTOBOND® + Supa Mega Microscope Slides, CellPath Ltd, Newtown, UK) suitable for IHC.

Specific individual odontocetes from the tissue archive were chosen based on recorded evidence of old age (worn/lost teeth, grossly appreciable relative increase in brain white matter compared with grey matter, age derived from histological examination of teeth or life‐long photo identification records) and well‐preserved tissue (minimal autolysis) and included 18 animals: Risso's dolphin (*n* = 2), long‐finned pilot whale (*Globicephala melas*) (*n* = 5), white‐beaked dolphin (*Lagenorhynchus albirostris*) (*n* = 5), harbour porpoise (*Phocoena phocoena*) (*n* = 5) and a single bottlenose dolphin (Table 1). Additionally, for each species that was found to be positive for Aβ peptide and/or pTau, a young adult or immature animal of that species was selected (*n* = 4, Table 1) from the tissue archive, and sections from two brain regions (see below) were subjected to identical IHC to determine if the chosen target proteins were present constitutively in that specific species of odontocete.

**TABLE 1:** Details of odontocetes selected and qualitative immunohistochemistry result of the initial two regions of cerebral cortex examined for amyloid‐beta peptide and hyperphosphorylated tau protein

ID	Species	Sex	Estimated age group	Probable cause of death	Initial Aβ IHC	Subsequent pTau IHC
Gg1	*Grampus griseus*	F	Adult	Peritonitis	Pos	NA
Gg2	*Grampus griseus*	F	Aged	Live stranding	Pos	NA
Gm1	*Globicephala melas*	F	Aged	Pneumonia	Pos	Pos
Gm2	*Globicephala melas*	M	Aged	Mass stranding	Pos	Neg
Gm3	*Globicephala melas*	F	Aged	Mass stranding	Pos	Pos
Gm4	*Globicephala melas*	F	Aged	Live stranding	Pos	NA
Gm5	*Globicephala melas*	M	Aged	Pneumonia	Pos	Pos
La1	*Lag. albirostris*	F	Adult	Unknown	Pos	NA
La2	*Lag. albirostris*	F	Aged	Unknown/old age	Pos	NA
La3	*Lag. albirostris*	M	Aged	Bacterial pneumonia	Pos	NA
La4	*Lag. albirostris*	M	Aged	Euthanized–LS	Pos	NA
La5	*Lag. albirostris*	M	Adult	Live stranding	Pos	Pos
Pp1	*Phocoena phocoena*	F	Aged	Unknown/old age	Pos	NA
Pp2	*Phocoena phocoena*	F	Aged	Gastritis	Pos	NA
Pp3	*Phocoena phocoena*	F	Aged	Unknown/old age	Pos	NA
Pp4	*Phocoena phocoena*	F	Aged	Live stranding	Pos	NA
Pp5	*Phocoena phocoena*	M	Aged	Old age/debilitation	Pos	NA
Tt1	*Tursiops truncatus*	M	>32 years	Pulmonary oedema	Pos	Pos
Gm6	*Globicephala melas*	F	Young adult	Live stranding	NA	NA
Gm7	*Globicephala melas*	M	Subadult	Live stranding	NA	NA
La6	*Lag. albirostris*	F	Subadult	Live stranding	NA	NA
Tt2	*Tursiops truncatus*	M	<1 year	Septicaemia	NA	NA

*Note*: The first 18 animals were the initial selection of older animals. The last four animals are the younger, control, animals that were not subjected to the initial immunohistochemistry screening. *Lag*. = *Lagenorhynchus*; F = female; M = male; LS = live‐stranded; IHC = immunohistochemistry; Aβ = amyloid‐beta peptide; pTau = hyperphosphorylated tau protein; Pos = positive; Neg = negative; NA = not applicable.

### 2.3 IHC

Initially, sections of two areas of the cerebral cortex (limbic and anterior paralimbic lobes where they overlay the basal ganglia and the supralimbic/paralimbic/limbic lobes where they overlay the thalamus), equivalent to the anatomical areas in human brains where APs accumulate initially in AD (Perl, 2021), were subjected to IHC for Aβ. If positive immunolabelling for Aβ revealed APs were present in either of these two regions, sections of all the brain regions available from the animal were subjected to IHC for Aβ. All sections from animals with APs in the initially screened two areas of cerebral cortex and a single animal with no APs present (Gm5) were subjected to IHC for pTau. Additionally, two sections of the cerebral cortices, from the same locations as those used for the initial screening for Aβ, from all the four young adult/immature animals were subjected to IHC for Aβ and pTau.

Immunolocalization of Aβ and APs was performed with rabbit monoclonal anti‐beta Amyloid 1‐42 antibody [mOC64] (ab201060, abcam, Cambridge, UK), specific for the human Aβ1‐42 conformation, diluted 1:25,000 in 2% normal goat serum (NGS) in phosphate‐buffered saline with 0.05% Tween20 added (PBST). To localize pTau, mouse monoclonal antibodies raised against Phospho‐Tau (Thr231) [AT180] (MN1040, ThermoFisher, Renfrew, UK), which labels pre‐NFTs and so would be expected to detect early‐stage AD‐like pathology, and antibodies raised against Phospho‐Tau (Ser202, Thr205) [AT8] (MN1020, ThermoFisher), which detects well‐developed NFTs present in later‐stage AD‐like pathology, were used, both diluted 1:1000 in 5% NGS/PBST and applied to separate semi‐serial sections.

Mounted brain tissue sections were dewaxed in xylene (2 × 2 min), rehydrated through graded alcohols (100% × 2 min, 90% × 2 min, 70% × 2 min) and then placed in running tap water (5 min) prior to immersion in 0.01 m sodium citrate solution, pH 6.0, and heated to 121°C for 10 min in an autoclave (Compact Benchtop Priorclave, London, UK) for heat‐induced epitope retrieval (HIER). Subsequently, slides were allowed to cool to 50°C and then placed in 70% alcohol. Endogenous tissue peroxidase activity was quenched by immersion in 3% H_2_O_2_ in methanol (v/v) for 30 min prior to washing in running tap water (5 min) then immersed in PBST (3 × 5 min). Non‐specific antibody binding was blocked by incubation with NGS diluted (2% for mOC64, 5% for the AT180 and AT8) in PBST for 1 h at room temperature. Slides were drained and the primary antibody was added (see above) and incubated overnight at 4°C in a moisture chamber. Slides were then drained and washed in PBST (3 × 5 min). Visualization of the primary antibody was by Agilent DAKO EnVision™ Detection Systems Peroxidase/DAB: Rabbit HRP kit (for mOC64) or Mouse HRP kit (for AT180 and AT8) (VECTOR laboratories LTD, Peterborough, UK) as per manufacturer's instructions. The chromogen, DAB (3,3′‐diaminobenzidine), was added to the slides for 6–8 min and then washed in running tap water (5 min); the sections were counterstained with haematoxylin (30 sec), followed by immersion in Scott's tap water substitute (a few seconds), washed in running tap water (5 min), dehydrated through graded alcohols, cleared in xylene and mounted.

To label glia by IHC, semi‐serial sections of brain tissue mounted on microscope slides from the same animals as above were dewaxed and rehydrated as above prior to placing into running tap water for 5 min. Sections were then subjected to citric acid (pH 6.0) pretreatment in a pressure cooker for 10 min for HIER and then washed in de‐ionized water for 5 min. Endogenous peroxidases were neutralized in Peroxidase block (RE7157, Leica Biosystems) for 30 min followed by 2 × 5 min washes in 1× tris‐buffered saline (TBS) (50 mM). Sections were incubated with Protein Block (RE7158, Leica Biosystems) for 15 min, to block non‐specific binding, followed by 2 × 5 min washes in TBS. Reactive astrocytes (anti‐GFAP: Z0334, diluted 1:800; Dako, Agilent Technologies LDA UK Limited, Stockport, UK) or microglia (anti‐Iba1: 019‐19741, diluted 1:750; Dako) were labelled with a respective primary antibody diluted in TBS for 30 min and then washed 2 × 5 min in TBS. Visualization of the primary antibodies was by the Novolink Polymer Detection Kit (RE7280‐K, Leica Biosystems, Milton Keynes, UK) according to manufacturer's instructions. Briefly, samples were treated with Post Primary (RE7159, Leica Biosystems) for 30 min, washed 2 × 5 min in TBS, and then incubated with Novolink Polymer (RE7161, Leica Biosystems) for 30 min. After a further 2 × 5 min washes in TBS, the peroxidase activity was developed with a 50:1 DAB substrate buffer (RE7163, Leica Biosystems): DAB Chromogen (RE7162, Leica Biosystems) solution for 4 min. Slides were then rinsed thoroughly in water, dehydrated through graded alcohols and xylene, and coverslips were applied using DPX Mountant (Sigma 06522). Slides were left to dry overnight before imaging. All labelling was performed at room temperature.

For immunofluorescence labelling, semi‐serial sections of brain tissue were dewaxed, rehydrated in ethanol and underwent HIER as for labelling of glia (see above). After rinsing in water for 5 min, sections were placed in blocking buffer (PBS, 3% Triton‐X 100, 10% normal donkey serum [NDS]) for 1 h at room temperature. Samples were then incubated in respective primary antibodies (diluted in blocking buffer) overnight at 4°C as follows: Aβ fibrillar oligomers: Rabbit mOC AB2286 (1:1000), astrocytes: Chicken GFAP ab4674 (1:1000), microglia: Goat Iba1 ab5076 (1:1000), all from abcam, UK, or Aβ fibrillar oligomers: Rabbit mOC AB2286 (1:1000), phospho‐tau AT8 MN1020 (ThermoFisher 1:1000). Samples were then washed 2 × 5 min in PBS and incubated with secondary antibodies: donkey anti‐rabbit‐594 (ThermoFisher A‐21207, 1:500), donkey anti‐chicken‐488 (Sigma SAB4600031, 1:250), donkey anti‐goat‐647 (ThermoFisher A‐21447, 1:500) or goat anti‐mouse IgG1‐594 (ThermoFisher A‐21125) and donkey‐anti‐rabbit IgG‐647 (ThermoFisher A‐32795) diluted in PBS for 1 h at room temperature in the dark. Slides were then washed 2 × 5 min in PBS. Sections labelled with AT8 and mOC were stained with 0.05% Thioflavin S in 50% ethanol for 8 min and washed in 80% ethanol then water. Coverslips were mounted with Epredia™ Immu‐Mount™ Medium (ThermoFisher). Slides were allowed to dry overnight, in the dark, prior to imaging.

All primary antibodies were titrated initially on human brain tissue from a definitively diagnosed AD case obtained from Edinburgh University's Brain and Tissue Bank (Edinburgh, UK) prior to examining the initial two sections from each chosen aged odontocete. Subsequently, antibodies were titrated on brain tissue sections from the odontocete that was found to have the most APs on the initial two sections examined (animal Tt1) for antibody mOC64 or the most pTau (animal Gm5) for antibodies AT180 and AT8.

Sections from these respective animals were used for positive control preparations for all further IHC. Methodology negative controls included substitution of primary antibodies with identical species and isotypes (rabbit IgG Isotype Control [ThermoFisher] for antibody mOC64 and mouse IgG1κ Isotype Control [P3.6.2.8.1], eBioscience™ [ThermoFisher] for antibodies AT180 and AT8). An additional negative control composed of omission of the primary antibodies, to ensure non‐specific binding of the secondary antibody in the visualization systems did not occur (Dagleish et al., 2010), was included also.

### 2.4 Imaging and scoring of IHC

Slides immunolabelled for amyloid and tau with antibodies mOC64, AT180 and AT8 were examined with an Olympus BX51 microscope and photomicrographs taken with an Olympus DP70 digital camera using analySIS® software (Soft Imaging System GmbH, Műnster, Germany). Each section was scored subjectively by two people (MCV and MPD) based on the amount of immunolabelling present: 0 = no labelling, 1 = minimal (<5% tissue labelled), 2 = small (>5–10%), 3 = medium (>10–30%), 4 = large (>30–60%) and 5 = very large amount (>60%).

Slides labelled for GFAP and Iba1 visualized with DAB were imaged on an AxioImager Z2 microscope (Zeiss) and images obtained with StereoInvestigator software (MicroBrightField). GFAP burdens were obtained by automated thresholding of DAB signal in StereoInvestigator software and calculation of the percent area occupied by positive labelling using ImageJ. Iba1 labelling was relatively weak in the odontocete brain sections examined, with suboptimal signal:noise ratio permitting only qualitative observations. Immunofluorescence labelled slides (for Aβ, GFAP and Iba1) were imaged using an oil immersion objective (×63) on TCS SP8 Confocal microscope (Leica).

## 3 RESULTS

### 3.1 Aβ and AP detection by IHC

**FIGURE 1:**
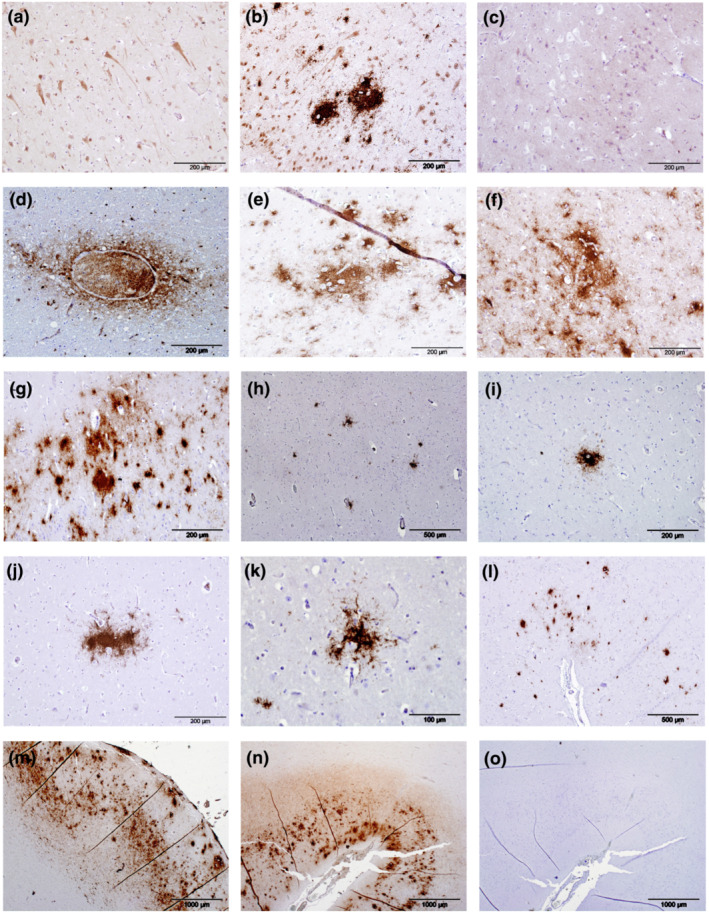
Immunolabelling (brown pigment) of amyloid‐beta peptide (Aβ) and amyloid plaques (APs) in the cerebrocortical grey matter of odontocetes. Aβ was present in the cell bodies of large neurons within the cerebrocortical grey matter and the labelling frequently extended into the axons (a, supralimbic/paralimbic/limbic lobes, animal Gg2). Intense labelling of Aβ was present in and around the nuclei in a small number of neurons with intensely labelled cytoplasm (b, limbic and anterior paralimbic lobes, animal Gm1). Some odontocetes had minimal intraneuronal labelling of Aβ represented by fine granular deposits within the cytoplasm (c, animal La1). Labelling of Aβ was present in and around a small number of the larger blood vessel (d, limbic and anterior paralimbic lobes, animal La5). When present, large deposits of Aβ in the neuropil in cerebrocortical layer I were diffuse, irregularly shaped with poorly defined borders with some coalescence forming APs present (e, supralimbic/paralimbic/limbic lobes, animal Tt1). When abundant, APs frequently coalesced (f, supralimbic/paralimbic/limbic lobes, animal Gm1; g, supralimbic/paralimbic/limbic lobes, animal Tt1). APs were distributed sparsely in animal La5 and did not coalesce (h, limbic and anterior paralimbic lobes). APs varied in shape and size within the same animal (i, limbic and anterior paralimbic lobes; j, supralimbic/paralimbic/limbic lobes; k, lingual lobe, all animal La5). Animal Gm1 had a medium number of APs present in the cerebrocortical grey matter, some of which had coalesced (l, supralimbic/paralimbic/limbic lobes). Animal Tt1 had the largest number of APs present, and these extended throughout all sections of cerebrocortical grey matter examined (m, supralimbic/paralimbic/limbic lobe). Positive control sections were composed of human brain tissue from a case of definitely diagnosed Alzheimer's disease (n). Sections used for negative controls were composed of semi‐serial sections of human brain tissue from a case of definitely diagnosed Alzheimer's disease (o) or positive odontocete brain tissue and were devoid of any immunolabelling

Examination of the initial two chosen areas of cerebral cortex from the 18 aged odontocetes showed all had intraneuronal cytoplasmic immunolabelling of Aβ (Table 1, Figures 1a–c), the extent of which varied greatly between animals. Intraneuronal Aβ labelling was restricted to large neurons within the cerebrocortical grey matter, forming areas composed of greatly differing numbers of labelled neurons surrounded by neurons devoid of labelling. When present, labelling was more notable in the cell body, but it frequently extended in to the axon (Figure 1a,b). A small to medium number of neurons showed intense labelling concentrated in and around the cell nucleus (Figure 1b). Most blood vessel contents were devoid of labelling, but intraluminal cells in a small number of the larger vessels showed positive labelling for Aβ, along with the neuroparenchyma directly adjacent to the vessel (Figure 1d). In the cerebrocortical neuropil, the large deposits of Aβ present in layer I were diffuse, irregularly shaped with poorly defined boarders and some were coalescing (Figure 1e), whereas accumulations of Aβ in the neuropil of cerebrocortical layer V labelled intensely and, in three animals (La5, Tt1 and Gm1), formed compact APs (Figure 1f,g). The presence of the blood vessel‐associated positive labelling was not linked to the presence of APs (see below) and was not present in any of the methodology negative controls.

Sections from all available brain regions of the three animals with APs (La5, Tt1 and Gm1) and one further animal (Gm5), which was negative for APs but had large amounts of pTau present (see below), were examined after re‐titration of the primary antibody in these sections and showed that Aβ was found predominantly in the cerebral cortex (Table 2). The basal ganglia/anterior corpus callosum and thalamus had only small amounts of Aβ‐positive labelling present (Table 2). A few small Aβ accumulations were observed in the midbrain in one animal (Tt1); otherwise, this region was devoid of labelling also. In all these four animals (La5, Tt1, Gm1 and Gm5), the pons/cerebellar peduncles, cerebellum, medulla and all white matter were devoid of any labelling for Aβ (Table 2). One animal (Gm5), which had minimal labelling of Aβ in the initial screening of two chosen brain regions and had no APs, had similar minimal labelling for Aβ and no APs when all brain regions available were examined (Table 2).

**TABLE 2:** Abundance and distribution of amyloid‐beta peptide (Aβ), detected by antibody mOC64, in all available sections of the brains of three odontocetes (Gm1, La5 and Tt1) with amyloid plaques present and one (Gm5) with no amyloid plaques present, and the two selected sections of cerebral cortex from the four younger, control, odontocetes (Gm6, Gm7, La6 and Tt2)

	Gm1	Gm5	La5	Tt1	Gm6	Gm7	La6	Tt2
Basal ganglia	2	0	1	3	NA	NA	NA	NA
Limbic and anterior paralimbic lobes of CC	3	1	2	4	0	1	1	1
Thalamus	1	0	1	2	NA	NA	NA	NA
Supralimbic/paralimbic/limbic lobes of CC	3	1	1	5	0	1	1	1
Lingual lobe of CC	0	1	1	5	NA	NA	NA	NA
Midbrain	0	0	0	1	NA	NA	NA	NA
Pons and cerebellar peduncles	0	0	0	0	NA	NA	NA	NA
Cerebellar vermis	0	0	0	0	NA	NA	NA	NA
Medulla	0	0	0	0	NA	NA	NA	NA

*Note*: CC = cerebral cortex; 0 = no labelling; 1 = minimal; 2 = small; 3 = medium; 4 = large; 5 = very large amount present; NA = not applicable.

The small number of APs that were present in the brain of the Atlantic white‐beaked dolphin (La5) were of various shapes and sizes and distributed sparsely throughout the neuroparenchyma of the cerebrocortical grey matter (Figure 1h,i–k). APs were more abundant and had coalesced in the long‐finned pilot whale Gm1 (Figure 1l) and were most numerous in the aged bottlenose dolphin (Tt1, Figure 1m). In the latter animal, despite being numerous, APs were restricted to areas that were highly Aβ‐positive (score ≥ 4) (Figure 1m,e–g, Table 2). Irrespective of frequency, all APs were restricted to the cerebrocortical grey matter and most frequent in neuronal layers I, III and V (Figure 1m,n). Labelling in the three animals with APs present (La5, Tt1 and Gm1) was consistent when compared with the two sections examined initially; the large deposits of Aβ present in cerebrocortical layer I were diffuse, irregularly shaped with poorly defined boarders and coalescing (Figure 1e), whereas accumulations of Aβ in cerebrocortical layer V labelled intensely, often forming compact APs (Figure 1f,g).

Of the four younger odontocetes, one (Gm6) was devoid of any labelling of Aβ but a small amount of intraneuronal labelling was present in the other three younger animals (Gm7, La6 and Tt2) in the sections of cerebral cortex examined (Table 2). The human brain tissue‐positive control, composed of sections from a definitely diagnosed case of AD, was consistently positive in the distribution expected as were the sections from the odontocete with the most APs that was used in each run of IHC as a positive control (Tt‐1, Figure 1n). All negative control preparations were devoid of any labelling (Figure 1o).

### 3.2 Phosphorylated tau changes

Six animals were subjected to IHC for tau phosphorylated at residue 231 in the two initially chosen brain regions, including the three animals that were positive for APs (Gm1, La5 and Tt1) and three animals without APs (Gm2, Gm3 and Gm5). Five animals (Gm1, Gm3, Gm5, La5 and Tt1) showed immunolabelling for pTau with antibody AT180 (Table 1), and this included all three animals that had well‐defined APs.

**FIGURE 2:**
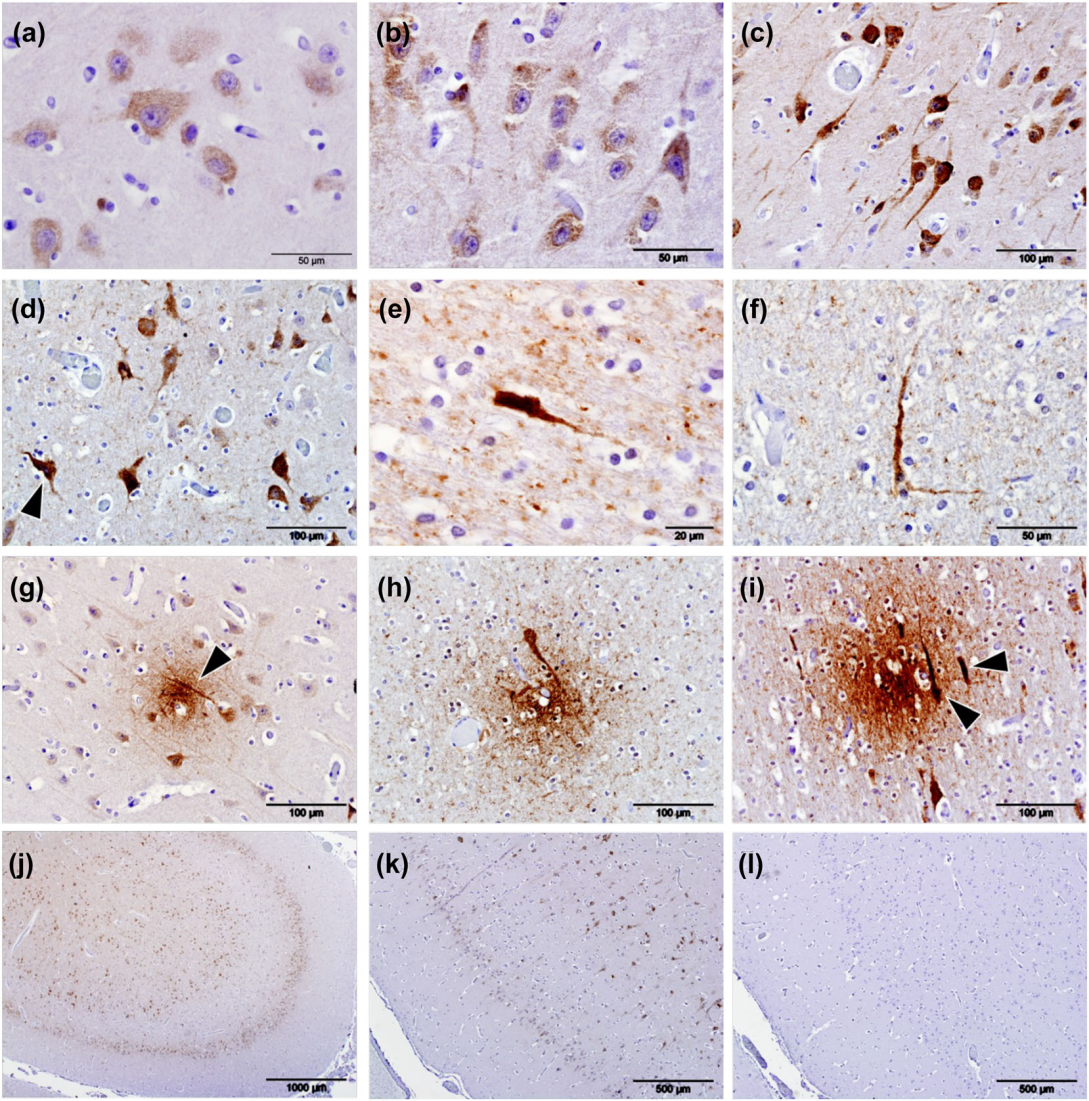
Immunolabelling (brown pigment) of phosphorylated tau protein (pTau) using antibody AT180 in the cerebral cortices of odontocetes. When at lower intensities, immunolabelling of pTau was present as cytoplasmic granules within the cell bodies of neurons (a), and with increased intensity of labelling, pTau frequently extended into the axons and dendrites (b,c). Irrespective of the intensity of cytoplasmic labelling, nuclei were consistently devoid of any labelling (c) although neurons that labelled intensely for pTau frequently contained pyknotic nuclei (d, arrowhead). Intensely phospho‐tau‐stained cells were found in animal (Gm5) and present in all sections of cerebral cortex available for examination (e,f). Neuritic plaques were present in both the white and grey matter in all four aged animals examined (Gm1, Gm5, La5 and Tt1) and within the grey matter often colocalized with neuropil threads (g, arrowhead). Similarly, neuritic plaques colocalized with phospho‐tau‐positive cell bodies (h,i, arrowheads). pTau labelling was present predominantly in cerebrocortical layers II and V (j). Positive control sections were composed of human brain tissue from a case of definitely diagnosed Alzheimer's disease (k). Semi‐serial sections were used for the controls (k, positive; l, negative control)

Sections of all available brain regions of the three animals positive for APs (Gm1, La5 and Tt1) and the animal without APs but with the most abundant pTau labelling in the two initial sections examined (Gm5) were investigated for the presence of pTau changes using antibody AT180. All three regions of the cerebral cortices examined contained more abundant labelling of pTau compared with the subcortical regions examined (Table 3). Labelling was widespread and primarily in the grey matter. Intracellular labelling was present within the cytoplasm of the cell bodies of larger neurons, sparing the nuclei (Figures 2a–c) and occasionally extended into the axons and dendrites (Figure 2b), the latter more frequently when labelling was intense (Figure 2c). When present in less intense accumulations, intracellular labelling was clearly granular (Figure 2a,b) but when intense, typically, obscured the detail of the cytoplasm of affected cells, and some of the more intensely labelled neurons had pyknotic nuclei (Figure 2d). Intensely labelled pTau inclusions resembling NFTs were present in animal Gm5 only (Figure 2e), along with numerous positively labelled NTs (Figure 2f,g). All four aged animals (Gm1, Gm5, La5 and Tt1) had positively labelled neuritic plaques in the cerebrocortical grey and white matter. The neuritic plaques within the grey matter of Gm5 often colocalized with NTs (Figure 2g) and pTau‐positive cells (Figure 2h,i) when present. Labelling of pTau was predominantly within neuronal layers II and V of the cerebrocortical grey matter, and approximately twice as much labelling of the larger neurons in layer V was present compared with the smaller pyramid‐like neurons in layer II (Figure 2j). The positive control was composed of human brain tissue sections from a definitely diagnosed case of AD and was consistently positive in the distribution expected, as were the sections from the odontocete with the most pTau labelling that was used in each run of IHC as a positive control (Gm5, Figure 2k). All negative control preparations were devoid of any labelling (Figure 2l).

**TABLE 3:** Abundance and distribution of phosphorylated tau protein (pTau), detected by antibody AT180, in all available sections of the brains of three odontocetes (Gm1, La5 and Tt1) with amyloid plaques present and one (Gm5) with no amyloid plaques present, and the two selected sections of cerebral cortex from the four younger, control, odontocetes (Gm6, Gm7, La6 and Tt2)

	Gm1	Gm5	La5	Tt1	Gm6	Gm7	La6	Tt2
Basal ganglia	0	0	0	0	NA	NA	NA	NA
Limbic and anterior paralimbic lobes of CC	2	5	2	3	1	0	1	5
Thalamus	2	1	1	1	NA	NA	NA	NA
Supralimbic/paralimbic/limbic lobes of CC	3	5	3	2	1	0	1	5
Lingual lobe of CC	3	5	1	3	NA	NA	NA	NA
Midbrain	0	0	0	0	NA	NA	NA	NA
Pons and cerebellar peduncles	0	0	0	0	NA	NA	NA	NA
Cerebellar vermis	0	0	0	0	NA	NA	NA	NA
Medulla	0	1	1	0	NA	NA	NA	NA

*Note*: CC = cerebral cortex; 0 = no labelling; 1 = minimal; 2 = small; 3 = medium; 4 = large; 5 = very large amount present; NA = not applicable.

**FIGURE 3:**
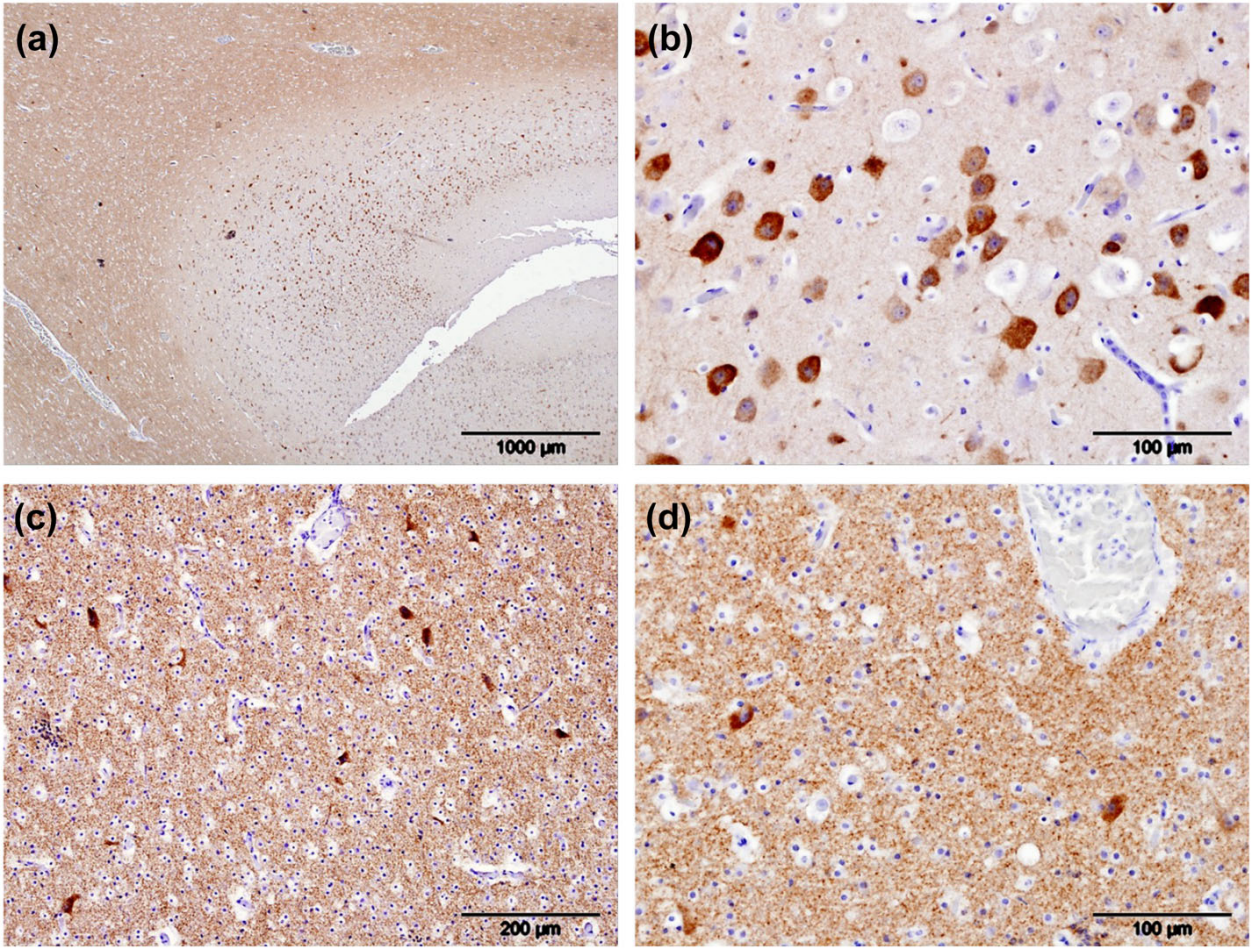
Phosphorylated tau (pTau) localized using antibody AT180 (brown pigment), in a young bottlenose dolphin (Tt2). pTau was present within neuropil of the white matter but not the grey matter (a). It was also present in the cytoplasm of nearly all neurons in the cerebrocortical grey matter but absent from the nuclei and surrounding neuropil (b). Furthermore, it was present within the neuropil of the white matter of the supralimbic/paralimbic/limbic lobes of the cortex and the cytoplasm of neurons contained within it (c). Greater magnification of cerebrocortical white matter highlighted the intense cytoplasmic labelling of neurons for pTau while sparing their nuclei (d)

Of the four young animals subjected to IHC for pTau in the two chosen regions of the cerebral cortex, Gm7 was devoid of labelling and Gm6 and La6 had only small islands of punctate cytoplasmic labelling within the neurons in cortical layer V (Table 3). In Tt2, most neurons throughout all layers of the cerebral cortex labelled for pTau, as did the neuroparenchyma of the white matter but not that of the grey matter (Table 2, Figure 3a). Intraneuronal labelling in this animal, although intense, was restricted to the cytoplasm and completely spared the nuclei (Figure 3b). Furthermore, the white matter showed labelling of pTau in both neuroparenchyma and neurons, again sparing nuclei (Figure 3c,d).

Antibody AT8 failed to label pTau by IHC in any of the odontocetes examined yet consistently labelled the human brain tissue from a definitely diagnosed case of AD in the distribution expected (Figure S1A). Sections labelled by immunofluorescence by antibody AT8 or stained with Thioflavin S revealed no NFT staining in any of the animals with AT180‐positive pTau accumulation, despite both showing clear labelling in an AD‐positive control section (Figure S1B), indicating that the pTau pathology in the animals in the study had not reached the stage of full‐blown NFT pathology.

### 3.3 Glial association with APs

**FIGURE 4:**
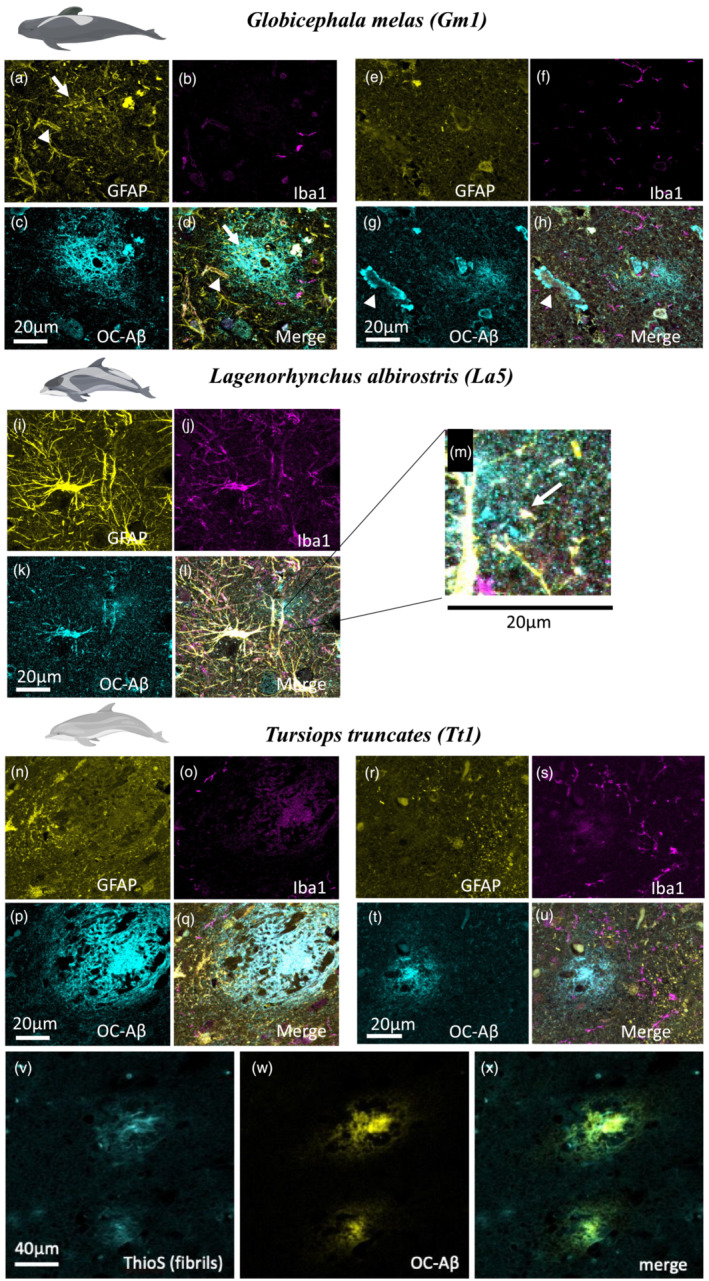
GFAP (yellow), Iba1 (magenta) and Aβ fibril (cyan) immunofluorescence labelling in *Globicephala melas* (Gm1) (a–h), *Lagenorhynchus albirostris* (La5) (i–m) and *Tursiops truncatus* (Tt1) (n–u). Gm1 shows diffuse plaques (d,h), evidence of CAA (g, arrowhead) and localized presence of astrocyte processes near some (a, arrow) but not all (e) plaques. Microglia are located near to, but not within plaques (b,f). La5 shows extremely strong GFAP labelling (i), which bled through to other channels. Small diffuse plaques were present, including next to blood vessels (zoomed in section part label m, arrow) but no strong evidence of plaque‐associated gliosis. Tt1 showed more punctate astrocyte labelling (n,r) with little association with plaques despite the presence of many diffuse (u) and the occasional dense‐core (q) plaques. Although there was no association of microglia with the dense‐core plaque (o), there were some microglial processes near the edge of a diffuse plaque (s). We confirmed dense‐core plaques contained fibrils using Thioflavin S staining (v), which appeared in amyloid plaques stained for Aβ (w,x). Scale bars = 20 or 40 μm as indicated on panels

Confocal immunofluorescence imaging was performed on each of the three animals that had positive IHC labelling for Aβ and pTau (Gm1, La5 and Tt1). Sections were assessed to determine the association of glial pathology with APs (Figure 4). As seen in the IHC (see above), most APs appeared diffuse, although a large, possibly dense‐core plaque was detected in Tt1 (Figure 4q). Staining with Thioflavin S to label beta‐pleated sheet fibrils confirms that APs identified as dense by morphology are fibrillar (Figure 4). Often, plaques were located near blood vessels, with some evidence of vascular Aβ accumulation indicative of cerebral amyloid angiopathy (CAA) (Figure 4h, arrowhead). Overall, few plaques were associated with glial processes in all three animals, although there were large differences in astrocyte morphology between animals. In Gm1, astrocytic endfeet were detected around blood vessels (Figure 4a,d, arrowhead), and astrocyte processes were detected within a minority of plaques (Figure 4a,d, arrow), with most plaques showing little glial accumulation (Figure 4h). Similarly, microglial processes were often detected near to, but not inside plaques (Figure 4b,f). Animal La5 showed intense GFAP labelling (Figure 4i) and association of Aβ with blood vessels (Figure 4m arrow) but little evidence of plaque‐associated gliosis, whereas animal Tt1 showed more punctate GFAP labelling (Figure 4n,r) with little evidence of major gliosis, even around dense plaques (Figure 4q). Sparse microglial processes were detected near the plaque boundary in some sections, but they did not infiltrate the centre of the plaque (Figure 4s).

### 3.4 Glial burden in grey and white matter

**FIGURE 5:**
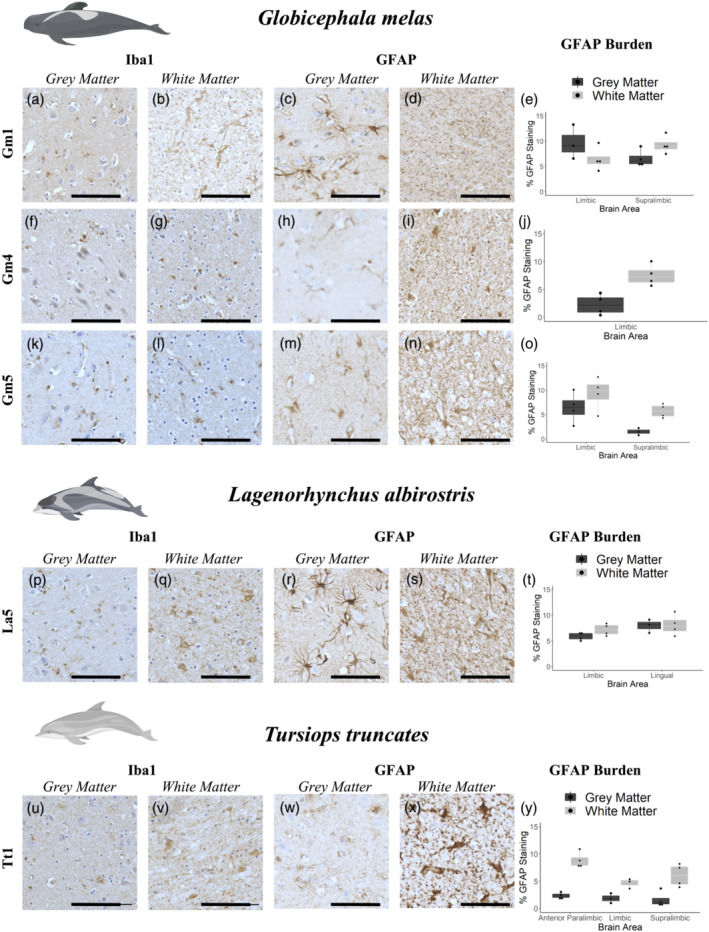
Immunohistochemical labelling (brown pigment) of astrocytes and microglia in grey and white matter from *Globicephala melas* (a–o), *Lagenorhynchus albirostris* (p–t) and *Tursiops truncatus* (u–y). All animals have both Aβ and pTau pathology as assessed by IHC, with the exception of Gm4 whose pTau status was not determined. Due to sample availability, different brain regions were assessed for each animal, with GFAP burden values are reported as % area of the image occupied by GFAP labelling. Graphs show an average ±SEM of 5 200 × 200 μm randomly selected regions of interest from both grey and white matter in each brain region (e,j,o,t,y). Iba1 labelling was fairly weak in all animals, although individual cells were resolvable in some sections, notably white matter from La5 (q) and Tt1 (v). GFAP labelling was clearer, with individual cells clearly resolvable in grey matter from Gm1 (c), Gm4 (h), Gm5 (m) and La5 (r). Tt1 showed a different pattern, with highly diffuse labelling in grey matter (w), but more readily resolvable cells in white matter (x), in contrast to the more punctate/mesh‐like GFAP labelling in white matter from other animals (d,i,n,s). Iba1 and GFAP labelling seemed uniformly distributed through grey/white matter, with little evidence of gliosis as may be seen around neuritic plaques in human Alzheimer's disease brain. Data are shown as box plots with each individual animal mean as data points. Scale bars = 100 μm

As immunofluorescence revealed considerable variation in glial morphology, further analysis of DAB‐stained labelling of glia was conducted. Gm1, Gm5, La5 and Tt1 (all Aβ‐ and pTau‐positive animals) and Gm4 (Aβ positive, but pTau status undetermined) were examined for GFAP and Iba1 burdens in grey and white matter (Figure 5). Iba1 labelling was weaker compared with immunofluorescence (Figure 4), with individual cells being difficult to resolve due to high levels of background labelling. Iba1 labelling was detected in both grey and white matter in all animals examined, with visually stronger labelling in La5 and Tt1 (particularly in the white matter, Figure 5q,v) than in any of the brains of Gm1, Gm4 or Gm5. GFAP labelling revealed similar glial morphology to that seen using immunofluorescence (Figure 4), with La5 showing intense astrocyte labelling in both grey and white matter (Figure 5r,s), whereas Tt1 had much more punctate labelling, particularly in grey matter where individual cells were hard to distinguish (Figure 5w). By contrast, individual cells were detectable in white matter in Tt1 (Figure 5x), unlike the labelling in Gm1, Gm4, Gm5 and La5, where astrocytes in the white matter formed more punctate, or mesh‐like networks (Figure 5d,i,n,s). Conversely, individually resolvable astrocytes were clearly visible in grey matter in these animals (Figure 5c,h,m,r). Whether this represents a species difference, random variation or differences in post‐mortem time intervals to tissue fixation requires further investigation. Astrocyte burdens (as measured by % GFAP labelling in each image) were between 2% and 10% in grey matter and 5–9% in white matter in *G. melas* (Gm) samples (Figure 5e,j,o). For La5, GFAP burden was between 6% and 8% in grey matter and 7–8% in white matter, depending on the brain region examined (Figure 5t). The highest difference between grey and white matter burdens was found in Tt1, with GFAP burden in grey matter ranging from 2% to 3% compared with 5–10% in white matter (Figure 5y). Both Iba1 and GFAP labelling seemed uniformly distributed through grey/white matter, with little evidence of gliosis as may be seen around neuritic plaques in human brains with AD. Further comparisons with younger animals to detect differences in global astrocyte burdens could be informative.

## 4 DISCUSSION

This is the first detailed investigation to determine if AD‐like pathology, as denoted by accumulations of APs and pTau, occurs in cetaceans, specifically odontocetes, and has shown the presence of both in three individual animals, each a different species. Additionally, the lesion distribution found in the brains of these odontocetes was analogous to the brain regions in humans most typically affected by AD.

Although odontocetes are typical representatives of the class Mammalia with the same genetic bauplan, during their secondary adaptation to that of an aquatic lifestyle, significant evolutionary changes in body structures and organs, including the brain, have resulted in profound modifications and considerable size progression (Glezer et al., 1988; Marino, 2004; Montgomery et al., 2013). Direct comparison between cetaceans and terrestrial mammals of the anatomical divisions of the telencephalic hemispheres into lobes is difficult, if not impossible, based solely on the location with respect to other structures in the rest of the brain less altered by evolution (Cozzi et al., 2017; Hof et al., 2005). The cetacean brains used in this study were taken for routine diagnostic evaluation of cause of death and trimmed at the Moredun Research Institute based on methods perfected for investigation of ruminants. Therefore, this is not wholly analogous when translated to odontocete brain anatomy, and future research should address this. Specifically, the lack of inclusion in these samples of the hippocampus, which is much reduced in cetaceans in terms of the classic anatomical ‘sea‐horse’ structure (Cozzi et al., 2017), is not sampled routinely for diagnostic purposes at present, but this will be addressed.

Our findings show that APs and, when present, the associated gliosis, along with accumulations of pTau in the dolphin brains examined, are similar in distribution to those found in pinnipeds (Takaichi et al, 2021), despite both studies investigating relatively small numbers of animals. Furthermore, our findings agree with those of Gunn‐Moore et al. (2018) with respect the distribution of APs and pTau in the cerebral cortices of striped dolphins but differ in the cerebellum, which was devoid of labelling for either of these targets in the present study. Although the distribution of APs present in aged dogs was similar, the variation in glial cell responses between the three different odontocete species made comparisons of this parameter impossible, and pTau was not evaluated (Davis et al., 2016; Rusbridge et al., 2018). In comparison with several species of non‐human primate that have been investigated, our findings are similar in that APs are present (Youssef et al., 2016). However, NFTs have not been reported in non‐human primates (Youssef et al., 2016), and although only one animal in the present study was positive for NFTs (Gm5 only, Figure 2e), given the relatively small number of odontocetes studies compared with non‐human primates, this may be significant.

In humans, AD‐associated APs are found initially in the basal parts of the frontal, temporal and occipital cortices and in advanced disease progresses to densely packed deposits in all cerebrocortical areas (Braak & Braak, 1991). The three odontocetes with APs present showed a similar pattern in that accumulations of Aβ were present in most regions of the cerebrocortical grey matter but APs were present predominantly in the rostro‐lateral cortices of the odontocete brain. This is known as the orbital lobe in odontocetes, contains the limbic and paralimbic lobes and is presumed the equivalent to the human frontal lobe (Cozzi et al., 2017). The cerebral cortex overlying the thalamus, which is composed of the supralimbic/paralimbic/limbic lobes in odontocetes, most likely translates to the parietal lobe in humans. Most APs detected were diffuse, located close to blood vessels, and lacked the overt gliosis typically associated with mature dense‐core neuritic plaques seen in human AD. In AD, Aβ accumulation is typically extracellular. However, we found nearly all individuals had intracytoplasmic labelling of cerebrocortical neurons in both the aged (*n* = 18/18) and younger animals (*n* = 3/4), irrespective of the presence or absence of APs. It is possible that this intracellular labelling was amyloid precursor protein (APP) and future studies would need to evaluate multiple antibodies, including those specific for APP, to determine this definitively if they can be validated in odontocete brain tissue. However, the presence of the APs in three odontocetes confirms the presence of, what is considered to be, a neurodegenerative lesion, although its significance in these species cannot be determined from this study alone.

Labelling of the perivascular neuropil of blood vessels was present in this study, and although this is a well‐documented in AD cases with CAA, it is typically associated with the shorter form of the peptide, Aβ40, which antibody mOC64 is not supposed to label (Reyes‐Ruiz et al., 2021). However, as the pathogenesis of Aβ accumulation in odontocetes is, as yet, unknown so antibody mOC64 may cross‐react with the shorter form of the Aβ in these species or the longer form may accumulate in the perivascular regions and further work using antibodies of known Aβ epitope, specificity is required to determine this possibility.

Phosphorylation of tau protein by various kinases is an important post‐translational modification allowing growth and remodelling of neurons. However, the phosphorylation of Thr231 is considered abnormal in healthy humans (Dávila‐Bouziguet et al., 2019) and an important event in the early neuronal pathogenesis of AD (Dávila‐Bouziguet et al., 2019; Neddens et al., 2018). In the odontocetes examined, pTau appeared as cytoplasmic granules within clusters of neurons present throughout the cerebral cortex which, in humans, denotes an early stage of NFT development (Augustinack et al., 2002). Furthermore, neurons that labelled intensely had pyknotic nuclei, typical of necrosis or apoptosis. The distribution of pTau in the different cerebrocortical layers in the odontocetes was similar to that present in AD‐associated pathology in humans (Pearson et al., 1985). Additionally, minimal intracytoplasmic granular labelling of pTau in the midbrain and medulla, as present in animal Gm5, can be present in humans with AD also (Dutschmann et al., 2010; Serrano‐Pozo et al., 2011). The most advanced neurofibrillary changes, including well‐defined neurofibrillary plaques, were found in animal Gm5 in which no APs were found. In humans, the presence of neurofibrillary changes in the absence of APs does not invariably lead to AD‐like neurodegeneration and cognitive decline (Braak & Braak, 1991), and this pathological presentation is not considered to be AD as both APs and pTau must be present, together with clinical cognitive dysfunctions, for a definitive diagnosis (Perl, 2021). The pTau labelling present in animal Tt2, the bottlenose dolphin calf, is different compared with all the other animals examined. The bacterium *Yersinia pseudotuberculosis* was recovered from this carcass, and histological examination resulted in a morphological diagnosis of minimal, acute, multifocal, non‐suppurative meningoencephalitis. Although this condition is not known to cause an increase in the amount of phosphorylation of tau (Thr231), some effect of the infection cannot be excluded so examination of multiple bottlenose dolphins and other odontocete calves might reveal whether these results where unique to this animal, due to the *Y. pseudotuberculosis* infection, or a constitutive occurrence of high levels of pTau in very young animals of this and other odontocete species.

The lack of labelling using the AT8 antibody may be due to several factors. Firstly, the epitope recognized by antibody AT8 (phosphorylated Ser202 and Thr205) is more associated with advanced extra‐neuronal tangles (Augustinack et al., 2002), and these are probably less likely to develop in wild, free‐ranging animals, even those in receipt of epimeletic behaviour. Secondly, the phosphorylation sites recognized by the antibody may not be conserved across humans and odontocetes, and further studies are required to determine this.

The earliest detection of NFTs in humans with AD is in the allocortical structures (Braak et al., 2006; Braak & Braak, 1991). Cetaceans have both an absolute and relatively small hippocampal formation and amygdala (Spocter et al., 2017a) with an extreme reduction in size in the paleocortex and archicortex (Cozzi et al., 2017). However, despite all four hippocampal regions being present (dentate gyrus, hippocampus, subiculum and entorhinal cortex), the size of the odontocete hippocampus is only 10% compared with that of humans (Berta et al., 2014; Oelschlager, 2008; Spocter et al., 2017b). Unfortunately, the hippocampal region was not available from any of the animals in this study as it is not identified and sampled routinely for examination during investigation into cause of death. Therefore, the neurofibrillary changes found in the odontocete brain samples could not be compared with those of the earliest appearance in the hippocampal region in humans with AD (Braak stages I and II) (Braak & Braak, 1991), but this is being addressed for future studies.

Accumulation of Aβ, APs and pTau in the brains of three elderly individual odontocetes, each a different species and restricted to brain regions analogous to humans with clinical AD, suggests these animals are susceptibility to spontaneous AD‐like pathology. However, for a definitive diagnosis of AD, an assessment of cognitive impairment is required also and this was not possible due to the source of the material. Accumulation of either solely APs or pTau does occur in humans, but neither is associated with AD. However, there are no records of simultaneous accumulation of the two proteins in the same individual in the absence of AD. Therefore, although it is tempting to speculate that the presence of the neuropathology in odontocetes is indicative of spontaneous clinical AD, this is not possible yet as AD‐like neuropathology can be present without symptoms or clinical signs of AD in humans (Braak & Braak, 1991). This is supported by the probability of a 78‐year‐old person being diagnosed with clinical AD being 5–10%, whereas the probability of finding significant AD‐related pathology in a person dying at 78 years old is 20–40% (Nelson et al., 2009).

Although PRLS has been proven in several odontocete species (Croft et al., 2017; Currie, et al., 2018; Ellis, Franks, Nattrass, Photopoulou et al., 2017; Foote, 2008; Marsh & Kasuya, 1968; Olesiuk et al., 1990), the reproductive behaviour and existence of an extended PRLS remain unknown in most species. Present data indicate that many odontocete species, including *G. melas*, do undergo an age‐related decline in fertility known as reproductive senescence (Ellis, Franks, Nattrass, Currie, et al., 2018). Conversely, no reproductive senescence has been found in *T. truncatus* (Ellis, Franks, Nattrass, Currie, et al., 2018), and free‐ranging pregnant females as old as 45 years were found despite females living, typically, no longer than 50 years (Reeves et al., 2002). We observed co‐occurring APs and phospho‐tau accumulation in this species, contradicting the hypothesis that AD, as defined by simultaneous accumulations of APs and pTau, is solely a consequence of PRLS, and the pathology could, therefore, be purely age related. This would explain the absence of this neuropathology in the *P. phocoena* and *G. griseus* examined as the former are a relatively short‐lived odontocete, approximately 24 years (Reeves et al., 2002), and usually live alone or in small groups of two to five animals, and the latter's life history, although estimated to live over 30 years (Reeves et al., 2002), remains elusive with little known definitively about lifespan or reproductive behaviour. For comparison, no signs of AD‐like neuropathology have been found in the African bush elephant (*Loxodonta africana*), a species that can reach an age of 60–70 years in the wild but does not undergo reproductive senescence (Lee et al., 2016). In future studies, it would be interesting to include mysticetes (baleen whales) as, although data are lacking, the known lifespans of all species exceed 50 years (53), with bowhead whales (*Balaena mysticetus*) possibly exceeding 200 years (Keane et al., 2015; Ma & Gladyshev, 2017).

Although typically descriptive and nonexperimental, over 100 reports of epimeletic behaviour have been documented in both captive and free‐ranging odontocetes. With just four exceptions, all cases involve species within the family of oceanic dolphins (Delphinidae) and 53% of all published reports refer to two genera: *Globicephala* and *Tursiops* (Bearzi et al., 2017), which include the species examined here. This may be due to the opportunistic character of the reports that are dependent upon species abundance, distribution and to what intensity the species is studied. However, it does indicate that these species would be able to aid the survival of sick or compromised conspecifics (Cockcroft & Sauer, 1990; Kuczaj et al., 2015). This would significantly affect the survival of animals with early cognitive dysfunctions thereby allowing the pathology to progress to a greater extent than would be expected in a less social or more solitary species.

A suggested hypothesis frequently quoted when cetacean mass stranding is the sick leader hypothesis (Mazzariol et al., 2018), though it is often difficult to find the leader of a pod and sometimes there are no obviously sick individuals in those that do strand. For resident killer whale pods, which are matrilineal family groups, post‐reproductive females lead collective movement, especially during periods of food scarcity (Brent et al., 2015). However, mass stranding of killer whales is extremely rare but, conversely, they are common in *G. melas*, and this species also lives in matrilineal family groups (Amos et al., 1991, 1993). In humans, the first symptoms of AD‐associated cognitive decline include confusion of time and place and a poor sense of direction. If the leader of a pod of *G. melas* suffered from a similar neurodegenerative‐related cognitive decline, this could lead to disorientation resulting in leading the pod into shallow water and subsequent stranding.

Like other dolphins, *T. truncatus* and *L. albirostris*, show tendencies to form social groups (Galatius et al., 2013; Galatius & Kinze, 2016), although the structure of these groups remains unknown. However, mass stranding of both these species are rare compared with *G. melas*.

## 5 CONCLUSION

Though humans are morphologically, physiologically and genetically more closely aligned to non‐human primates, odontocetes may be a more accurate, spontaneous model for studying AD, as non‐human primates do not develop AD spontaneously (Walker & Jucker, 2017; Youssef et al., 2016). The hypothesis that shared traits, such as extended PRLS and epimeletic behaviour, may be better indicators of susceptibility to aging‐associated disorders is not convincingly supported by our findings. However, the presence of APs and neurofibrillary changes in three different species of odontocetes is highly suggestive of some degree of equivalence to the AD‐like pathology found in humans. Further research should include the examination of more individuals, including sampling the hippocampus, different odontocete species and age groups and the inclusion of mysticetes and captive odontocetes with detailed known life histories. Furthermore, it would be possible and extremely valuable to investigate cognitive decline in captive *T. truncatus*, given our findings that they develop AD‐like pathology, and this would be eminently possible in the cohort kept by the US Navy given the training they receive and the complex tasks they perform (US Navy, 2022). Apart from the latter cohort, odontocetes are not going to be kept as experimental animals. However, determining the similarities and differences between human and odontocete neuropathology in comparison with incomplete and transgenic models might provide greater understanding of the pathogenesis, risk factors and underlying mechanisms of AD.

## CONFLICTS OF INTEREST

The authors declare that the research was conducted in the absence of any commercial or financial relationships that could be construed as a potential conflict of interest.

## AUTHOR CONTRIBUTIONS

MCV performed the IHC and histological evaluation of the sections, analysed the data and wrote the initial draft of the manuscript; CSD, JR and TLS‐J all performed IHC/immunofluorescence/imaging, interpreted the results, read and edited the manuscript; AJH interpreted the results, read and edited the manuscript; FG‐M co‐conceived the study (with MPD), recruited and supervised MCV, read and edited the manuscript; MPD co‐conceived the study (with FG‐M), performed the IHC and histological evaluation of the sections, directed and supervised MCV throughout the study and wrote and edited the manuscript.

6

### PEER REVIEW

The peer review history for this article is available at https://publons.com/publon/10.1111/ejn.15900.

## Supporting information


**Figure S1.** **A:** Human brain from a patient definitively diagnosed with Alzheimer's disease used as a positive control when labelling for pTau (brown pigment) using antibody AT8. All odontocetes examined failed to label with antibody AT8 despite a positive control section (as above) being included at all times. **B:** AT8 staining of phospho‐tau (magenta) was not observed in any of the animals in the study (example from animal Tt1 shown top row). Dense cored amyloid plaques labelled with both Aβ antibody OC (yellow) Thioflavin S to stain fibrils (Thioflavin S, grey) were observed (arrowheads). Both AT8 positive Thioflavin S positive fibrillar neurofibrillary tangles (arrows) and dense core plaques were observed in a positive control slide from an Alzheimer's case (bottom). Scale bar 40 μm.

## Data Availability

All micrographs published in this study are freely available on the University of Edinburgh DataShare platform https://datashare.ed.ac.uk/handle/10283/3076, and all images obtained during the study are available upon reasonable request from the corresponding author.

## References

[ejn15900-bib-0001] Alzheimer's Association . (2015). Alzheimer's Association report: 2015 Alzheimer's disease facts and figures. Alzheimer's & Dementia, 11, 332–384. 10.1016/j.jalz.2015.02.003 25984581

[ejn15900-bib-0002] Amos, B. , Barrett, J. , & Dover, G. A. (1991). Breeding behaviour of pilot whales revealed by DNA fingerprinting. Heredity, 67, 49–55. 10.1038/hdy.1991.64 1917551

[ejn15900-bib-0003] Amos, B. , Schlotterer, C. , & Tautz, D. (1993). Social structure of pilot whales revealed by analytical DNA profiling. Science, 260, 670–672. 10.1126/science.8480176 8480176

[ejn15900-bib-0004] Augustinack, J. C. , Schneider, A. , Mandelkow, E. M. , & Hyman, B. T. (2002). Specific tau phosphorylation sites correlate with severity of neuronal cytopathology in Alzheimer's disease. Acta Neuropathologica (Berl.), 103, 26–35. 10.1007/s004010100423 11837744

[ejn15900-bib-0005] Austad, S. N. , Hoffman, J. M. (2018) Is antagonistic pleiotropy ubiquitous in aging biology? Evolution, Medicine, and Public Health 2018 (1), 287–294. 10.1093/emph/eoy033 PMC6276058 30524730

[ejn15900-bib-0006] Bearzi, G. , Lavinia, E. , Piwetz, S. , Reggente, M. A. L. , & Cozzi, B. (2017). Cetacean behavior toward the dead and dying. In J. Vonk & T. Shackelford (Eds.). Encyclopedia of animal cognition and behavior. Springer. 10.1007/978-3-319-47829-6_2023-1

[ejn15900-bib-0007] Berta, A. , Ekdale, E. G. , & Cranford, T. W. (2014). Review of the cetacean nose: Form, function, and evolution. The Anatomical Record, 297, 2205–2215. 10.1002/ar.23034 25312374

[ejn15900-bib-0008] Braak, H. , Alafuzoff, I. , Arzberger, T. , Kretzschmar, H. , & Tredici, K. (2006). Staging of Alzheimer disease‐associated neurofibrillary pathology using paraffin sections and immunocytochemistry. Acta Neuropathologica (Berl.), 112, 389–404. 10.1007/s00401-006-0127-z 16906426 PMC3906709

[ejn15900-bib-0009] Braak, H. , & Braak, E. (1991). Neuropathological stageing of Alzheimer‐related changes. Acta Neuropathologica (Berl.), 82, 239–259. 10.1007/BF00308809 1759558

[ejn15900-bib-0010] Brent, L. J. N. , Franks, D. W. , Foster, E. A. , Balcomb, K. C. , Cant, M. A. , & Croft, D. P. (2015). Report ecological knowledge, leadership, and the evolution of menopause in killer whales. Current Biology, 25, 746–750. 10.1016/j.cub.2015.01.037 25754636

[ejn15900-bib-0011] Bufill, E. , Blesa, R. , & Agustí, J. (2013). Alzheimer's disease: An evolutionary approach. Journal of Anthropological Sciences, 91, 135–157. 10.4436/jass.91001 23579031

[ejn15900-bib-0012] Cipriani, G. , Dolciotti, C. , Picchi, L. , & Bonuccelli, U. (2011). Alzheimer and his disease: A brief history. Neurological Sciences, 32, 275–279. 10.1007/s10072-010-0454-7 21153601

[ejn15900-bib-0013] Cockcroft, V. G. , & Sauer, W. (1990). Observed and inferred epimeletic (nurturant) behaviour in bottlenose dolphins. Aquatic Mammals, 16(1), 31–32.

[ejn15900-bib-0014] Cozzi, B. , Huggenberger, S. , & Oelschlager, H. (2017). Brain, spinal cord, and cranial nerves. In Anatomy of dolphins insights into body structure and function (pp. 197–300). Academic Press. 10.1016/B978-0-12-407229-9.00006-3

[ejn15900-bib-0015] Croft, D. P. , Johnstone, R. A. , Ellis, S. , Nattrass, S. , Franks, D. W. , Brent, L. J. , Mazzi, S. , Balcomb, K. C. , Ford, J. K. , & Cant, M. A. (2017). Reproductive conflict and the evolution of menopause in killer whales. Current Biology, 27, 298–304. 10.1016/j.cub.2016.12.015 28089514

[ejn15900-bib-0016] Dagleish, M. P. , Benavides, J. , & Chianini, F. (2010). Immunohistochemical diagnosis of infectious diseases of sheep. Small Ruminant Research, 92, 19–35. 10.1016/j.smallrumres.2010.04.003

[ejn15900-bib-0017] Dávila‐Bouziguet, E. , Targa‐Fabra, G. , Ávila, J. , Soriano, E. , & Pascual, M. (2019). Differential accumulation of tau phosphorylated at residues Thr231, Ser262 and Thr205 in hippocampal interneurons and its modulation by tau mutations (VLW) and amyloid‐β peptide. Neurobiology of Disease, 125, 232–244. 10.1016/j.nbd.2018.12.006 30553886

[ejn15900-bib-0018] Davis, P. R. , Giannini, G. , Rudolph, K. , Calloway, N. , Royer, C. M. , Beckett, T. L. , Murphy, M. P. , Bresch, F. , Pagani, D. , Platt, T. , Wang, X. , Donovan, A. S. , Sudduth, T. L. , Lou, W. , Abner, E. , Kryscio, R. , Wilcock, D. M. , Barrett, E. G. , & Head, E. (2016). Aβ vaccination in combination with behavioral enrichment in aged beagles: Effects on cognition, Aβ, and microhemorrhages. Neurobiology of Aging, 49, 86–99. 10.1016/j.neurobiolaging.2016.09.007 27776266 PMC5154836

[ejn15900-bib-0019] De Waal, F. B. M. , & Preston, S. D. (2017). Mammalian empathy: Behavioural manifestations and neural basis. Nature Reviews. Neuroscience, 18, 498–509. 10.1038/nrn.2017.72 28655877

[ejn15900-bib-0020] Dutschmann, M. , Menuet, C. , Stettner, G. M. , Gestreau, C. , Borghgraef, P. , Devijver, H. , Gielis, L. , Hilaire, G. , & Van Leuven, F. (2010). Upper airway dysfunction of tau‐P301L mice correlates with tauopathy in midbrain and ponto‐medullary brainstem nuclei. The Journal of Neuroscience, 30, 1810–1821. 10.1523/JNEUROSCI.5261-09.2010 20130190 PMC6633985

[ejn15900-bib-0021] Ellis, S. , Franks, D. W. , Nattrass, S. , Cant, M. A. , Bradley, D. L. , Giles, D. , Balcomb, K. C. , & Croft, D. P. (2018). Postreproductive lifespans are rare in mammals. Ecology and Evolution, 8, 2482–2494. 10.1002/ece3.3856 29531669 PMC5838047

[ejn15900-bib-0022] Ellis, S. , Franks, D. W. , Nattrass, S. , Currie, T. E. , Cant, M. A. , Giles, D. , Balcomb, K. C. , & Croft, D. P. (2018). Analyses of ovarian activity reveal repeated evolution of post‐reproductive lifespans in toothed whales. Scientific Reports, 8(12833), 1–10. 30150784 10.1038/s41598-018-31047-8 PMC6110730

[ejn15900-bib-0023] Foote, A. D. (2008). Mortality rate acceleration and post‐reproductive lifespan in matrilineal whale species. Biology Letters, 4, 189–191. 10.1098/rsbl.2008.0006 18252662 PMC2429943

[ejn15900-bib-0024] Galatius, A. , Jansen, O. E. , & Kinze, C. C. (2013). Parameters of growth and reproduction of white‐beaked dolphins (*Lagenorhynchus albirostris*) from the North Sea. Marine Mammal Science, 29, 348–355. 10.1111/j.1748-7692.2012.00568.x

[ejn15900-bib-0025] Galatius, A. , & Kinze, C. C. (2016). *Lagenorhynchus albirostris* (Cetacea: Delphinidae). Mammalian Species, 48, 35–47. 10.1093/mspecies/sew003

[ejn15900-bib-0026] Glezer, I. I. , Jacobs, M. S. , & Morgane, P. J. (1988). Implications of the “initial brain” concept for brain evolution in Cetacea. The Behavioral and Brain Sciences, 11, 75–89. 10.1017/S0140525X0005281X

[ejn15900-bib-0027] Gunn‐Moore, D. , Kaidanovich‐Beilin, O. , Gallego Iradi, M. C. , Gunn‐Moore, F. , & Lovestone, S. (2018). Alzheimer's disease in humans and other animals: A consequence of postreproductive life span and longevity rather than aging. Alzheimer's & Dementia, 14, 195–204. 10.1016/j.jalz.2017.08.014 28972881

[ejn15900-bib-0028] Hashimoto, M. , Ho, G. , Takamatsu, Y. , Shimizu, Y. , Sugama, S. , Takenouchi, T. , Waragai, M. , & Masliah, E. (2018). Evolvability and neurodegenerative disease: Antagonistic pleiotropy phenomena derived from amyloid aggregates. Journal of Parkinson's Disease, 8, 405–408. 10.3233/JPD-181365 PMC6130413 30010144

[ejn15900-bib-0029] Hof, P. E. , Chanis, R. , & Marino, L. (2005). Cortical complexity in cetacean brains. The Anatomical Record – Part a Discoveries in Molecular in Cellular and Evolutionary and Biology, 287, 1142–1152. 10.1002/ar.a.20258 16200644

[ejn15900-bib-0030] Ittner, A. , Chua, S. W. , Bertz, J. , Volkerling, A. , van der Hoven, J. , Gladbach, A. , Przybyla, M. , Bi, M. , van Hummel, A. , Stevens, C. H. , Ippati, S. , Suh, L. S. , Macmillan, A. , Sutherland, G. , Kril, J. J. , Silva, A. P. , Mackay, J. P. , Poljak, A. , Delerue, F. , … Ittner, L. M. (2017). Site‐specific phosphorylation of tau inhibits amyloid‐beta toxicity in Alzheimer's mice. Science, 354, 904–908. 10.1126/science.aah6205 27856911

[ejn15900-bib-0031] Keane, M. , Keane, M. , Semeiks, J. , Webb, A. E. , Li, Y. I. , Quesada, V. , Craig, T. , Madsen, L. B. , van Dam, S. , Brawand, D. , Marques, P. I. , & Michalak, P. (2015). Insights into the evolution of longevity from the bowhead whale genome. Cell Reports, 10, 112–122. 10.1016/j.celrep.2014.12.008 25565328 PMC4536333

[ejn15900-bib-0032] Kent, S. A. , Spires‐Jones, T. L. , & Durrant, C. S. (2020). The physiological roles of tau and Aβ: Implications for Alzheimer's disease pathology and therapeutics. Acta neuropathologica (Berl.), 140(4), 417–447. 10.1007/s00401-020-02196-w 32728795 PMC7498448

[ejn15900-bib-0033] Kuczaj, S. A. , Frick, E. E. , Jones, B. L. , Lea, J. S. , Beecham, D. , & Schnöller, F. (2015). Underwater observations of dolphin reactions to a distressed conspecific. Learning & Behavior, 43, 289–300. 10.3758/s13420-015-0179-9 25898942

[ejn15900-bib-0034] Lee, P. C. , Fishlock, V. , Webber, C. E. , & Moss, C. J. (2016). The reproductive advantages of a long life: Longevity and senescence in wild female African elephants. Behavioral Ecology and Sociobiology, 70, 337–345. 10.1007/s00265-015-2051-5 26900212 PMC4748003

[ejn15900-bib-0035] Ma, S. , & Gladyshev, V. N. (2017). Molecular signatures of longevity: Insights from cross‐species comparative studies. Seminars in Cell & Developmental Biology, 70, 190–203. 10.1016/j.semcdb.2017.08.007 28800931 PMC5807068

[ejn15900-bib-0036] Marino, L. (2004). Cetacean brain evolution: Multiplication generates complexity. International Journal of Comparative Psychology, 17, 1–16. 10.46867/IJCP.2004.17.01.06

[ejn15900-bib-0037] Marsh, H. , & Kasuya, T. (1968). Evidence for reproductive senescence in female cetaceans. Report of the International Whaling Commission, 8, 57–74.

[ejn15900-bib-0038] Mazzariol, S. , Centellegheet, C. , Cozzi, B. , Povinelli, M. , Marcer, F. , Ferri, N. , Di Francesco, G. , Badagliacca, P. , Profeta, F. , Olivieri, V. , Guccione, S. , Cocumelli, C. , Terracciano, G. , Troiano, P. , Beverelli, M. , Garibaldi, F. , Podestà, M. , Marsili, L. , Fossi, M. C. , … Di Guardo, G. (2018). Multidisciplinary studies on a mass stranding of sperm whales (*Physeter macrocephalus*) along the Adriatic coast of Italy. Scientific Reports, 8(1), 11577. 10.1038/s41598-018-29966-7 30068967 PMC6070578

[ejn15900-bib-0039] Montgomery, S. H. , Geisler, J. H. , McGowen, M. R. , Fox, C. , Marino, L. , & Gatesyet, J. (2013). The evolutionary history of cetacean brain and body size. Evolution, 67, 3339–3353. 10.1111/evo.12197 24152011

[ejn15900-bib-0040] Mufson, E. J. , Counts, S. E. , Perez, S. E. , & Ginsberg, S. D. (2008). Cholinergic system during the progression of Alzheimer's disease: Therapeutic implications. Expert Review of Neurotherapeutics, 8, 1703–1718. 10.1586/14737175.8.11.1703 18986241 PMC2631573

[ejn15900-bib-0041] Neddens, J. , Temmel, M. , Flunkert, S. , Kerschbaumer, B. , Hoeller, C. , Loeffler, T. , Niederkofler, V. , Daum, G. , Attems, J. , & Hutter‐Paier, B. (2018). Phosphorylation of different tau sites during progression of Alzheimer's disease. Acta Neuropathologica Communications, 6, 1, 52–15. 10.1186/s40478-018-0557-6 29958544 PMC6027763

[ejn15900-bib-0042] Nelson, P. T. , Braak, H. , & Markesbery, W. R. (2009). Neuropathology and cognitive impairment in Alzheimer disease: A complex but coherent relationship. Journal of Neuropathology and Experimental Neurology, 68, 1–14. 10.1097/NEN.0b013e3181919a48 19104448 PMC2692822

[ejn15900-bib-0043] Oelschlager, H. H. A. (2008). The dolphin brain—A challenge for synthetic neurobiology. Brain Research Bulletin, 75, 450–459. 10.1016/j.brainresbull.2007.10.051 18331914

[ejn15900-bib-0044] Olesiuk, O. F. , Bigg, M. A. , & Ellis, G. M. (1990). Life history and population dynamics of resident killer whale (*Orcinus orca*) in the coastal waters of British Columbia and Washington State. Report of the International Whaling Commission, Special Issue, 12, 209–243.

[ejn15900-bib-0045] Orta‐Salazar, E. , Vargas‐Rodríguez, I. , Castro‐Chavira, S. A. , Feria‐Velasco, A. I. , & Díaz‐Cintra, S. (2016). Alzheimer's disease: From animal models to the human syndrome. In Update on dementia (pp. 191–224. ISBN 978‐953‐51‐2655‐3). IntechOpen. 10.5772/64619

[ejn15900-bib-0046] Parihar, M. S. , & Brewer, G. J. (2010). Amyloid beta as a modulator of synaptic plasticity. Journal of Alzheimer's Disease, 22, 741–763. 10.3233/JAD-2010-101020 PMC3079354 20847424

[ejn15900-bib-0047] Pearson, H. A. , & Peers, C. (2006). Physiological roles for amyloid β peptides. The Journal of Physiology, 575, 5–10. 10.1113/jphysiol.2006.111203 16809372 PMC1819417

[ejn15900-bib-0048] Pearson, R. C. A. , Esiri, M. M. , Hiorns, R. W. , Wilcock, G. K. , & Powell, T. P. S. (1985). Anatomical correlates of the distribution of the pathological changes in the neocortex in Alzheimer disease. PNAS, 82, 4531–4534. 10.1073/pnas.82.13.4531 3859874 PMC391136

[ejn15900-bib-0049] Perl, D. P. (2021). Neuropathology of Alzheimer's disease. Mount Sinai Journal of Medicine, 77, 32–42. 10.1002/msj.20157 PMC2918894 20101720

[ejn15900-bib-0050] Photopoulou, T. , Ferreira, I. M. , Best, P. B. , Kasuya, T. , & Marsh, H. (2017). Evidence for a postreproductive phase in female false killer whales *Pseudorca crassidens* . Frontiers in Zoology, 14, 1, 30–14. 10.1186/s12983-017-0208-y 28649267 PMC5479012

[ejn15900-bib-0051] Prince, M. , Knapp, M. , Guerchet, M. , McCrone, P. , Prina, M. , Comas‐Herrera, A. , Wittenberg, R. , Adelaja, B. , Hu, B. , King, D. , & Rehill, A. (2014). Dementia UK: Second edition—Overview. Alzheimer's Society.

[ejn15900-bib-0052] Reeves, R. R. , Stewart, B. S. , Clapham, P. J. , & Powell, J. A. (2002). Sea mammals of the world. First edit A &C Black Publishers Ltd.

[ejn15900-bib-0053] Reyes‐Ruiz, J. M. , Nakajima, R. , Baghallab, I. , Goldschmidt, L. , Sosna, J. , Mai Ho, P. N. , Kumosani, T. , Felgner, P. L. , & Glabe, C. (2021). An “epitomic” analysis of the specificity of conformation‐dependent, anti‐Aß amyloid monoclonal antibodies. The Journal of Biological Chemistry, 296, 100168. 10.1074/jbc.RA120.015501 33298522 PMC7949048

[ejn15900-bib-0054] Rusbridge, C. , Salguero, F. J. , David, M. A. , Faller, K. M. E. , Bras, J. T. , Guerreiro, R. J. , Richard‐Londt, A. C. , Grainger, D. , Head, E. , Brandner, S. G. P. , Summers, B. , Hardy, J. , & Tayebi, M. (2018). An aged canid with behavioral deficits exhibits blood and cerebrospinal fluid amyloid beta oligomers. Frontiers in Aging Neuroscience, 10, 1–8. 10.3389/fnagi.2018.00007 29441010 PMC5797595

[ejn15900-bib-0055] Sacchini, S. , Díaz‐Delgado, J. , Espinosa de Los Monteros, A. , Paz, Y. , Bernaldo de Quirós, Y. , Sierra, E. , Arbelo, M. , Herráez. P. , Fernández, A. (2020) Amyloid‐beta peptide and phosphorylated tau in the frontopolar cerebral cortex and in the cerebellum of toothed whales: Aging versus hypoxia. Biol Open **5**, 9(11):bio054734. 10.1242/bio.054734 33037014 PMC7657478

[ejn15900-bib-0056] Serrano‐Pozo, A. , Frosch, M. P. , Masliah, E. , & Hyman, B. T. (2011). Neuropathological alterations in Alzheimer disease. Cold Spring Harbor Perspectives in Medicine, 1, 1–23. 10.1101/cshperspect.a006189 PMC3234452 22229116

[ejn15900-bib-0057] Spocter, M. A. , Patzke, N. , & Manger, P. (2017a). Cetacean brains. In The curated reference collection in neuroscience and biobehavioral psychology. Springer. 10.1016/B978-0-12-809324-5.02175-1

[ejn15900-bib-0058] Spocter, M. A. , Patzke, N. , & Manger, P. R. (2017b). Cetacean brains. Reference Module in Neuroscience and Biobehavioral Psychology. 10.1016/B978-0-12-809324-5.02175-1

[ejn15900-bib-0059] Stoothoff, W. H. , & Johnson, G. V. W. (2005). Tau phosphorylation: Physiological and pathological consequences. Biochimica et Biophysica Acta, Molecular Basis of Disease, 1739, 280–297. 10.1016/j.bbadis.2004.06.017 15615646

[ejn15900-bib-0060] Stylianaki, I. , Komnenou, A. T. , Posantzis, D. , Nikolaou, K. , & Papaioannou, N. (2019). Alzheimer's disease‐like pathological lesions in an aged bottlenose dolphin (*Tursiops truncatus*). Veterinary Record Case Reports, 7, e000700. 10.1136/vetreccr-2018-000700

[ejn15900-bib-0061] Takaichi, Y. , Chambers, J. K. , Takahashi, K. , Soeda, Y. , Koike, R. , Katsumata, E. , Kita, C. , Matsuda, F. , Haritani, M. , Takashima, A. , & Nakayama, H. (2021). Amyloid β and tau pathology in brains of aged pinniped species (sea lion, seal, and walrus). Acta Neuropathologica Communications, 9, 1–15. 33413691 10.1186/s40478-020-01104-3 PMC7792306

[ejn15900-bib-0062] US Navy . (2022) Marine Mammal Program. Naval Information Warfare Centre Pacific https://www.niwcpacific.navy.mil/marine-mammal-program [Accessed May 2022].

[ejn15900-bib-0063] Walker, L. C. , & Jucker, M. (2017). The exceptional vulnerability of humans to Alzheimer's disease. Trends in Molecular Medicine, 23, 534–545. 10.1016/j.molmed.2017.04.001 28483344 PMC5521004

[ejn15900-bib-0064] Whitehouse, P. J. , Price, D. L. , Clark, A. W. , Coyle, J. T. , & DeLong, M. R. (2004). Alzheimer disease: Evidence for selective loss of cholinergic neurons in the nucleus basalis. Annals of Neurology, 10, 122–126. 10.1002/ana.410100203 7283399

[ejn15900-bib-0065] Youssef, S. A. , Capucchio, M. T. , Rofina, J. E. , & Chambers, J. K. (2016). Pathology of the aging brain in domestic and laboratory animals, and animal models of human neurodegenerative diseases. Veterinary Pathology, 53, 327–348. 10.1177/0300985815623997 26869150

