# Supplementary figures and images for: Alzheimer's disease‐like neuropathology in three species of oceanic dolphin
Source: Eur J Neurosci. 2022 Dec 27;57(7):1161–79. doi: 10.1111/ejn.15900 (PMC10947196; doi:10.1111/ejn.15900)

A

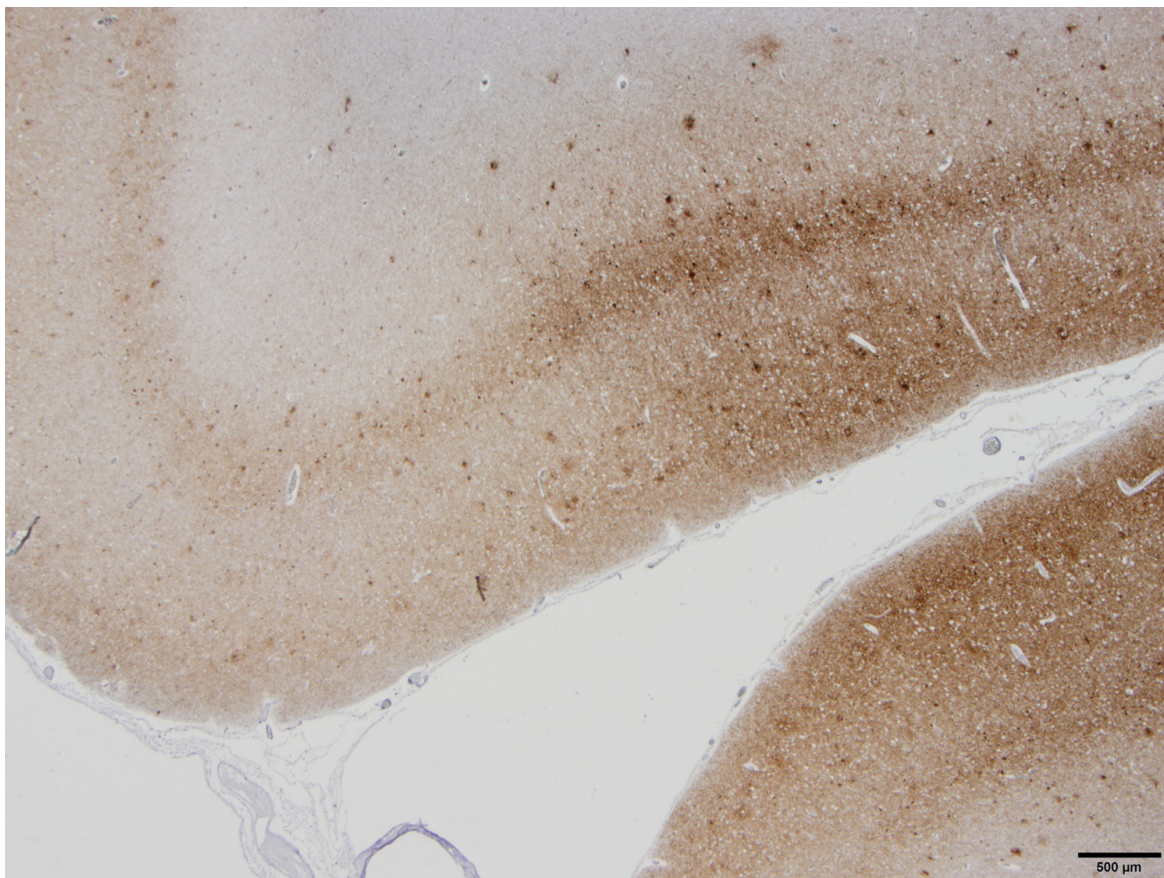

B

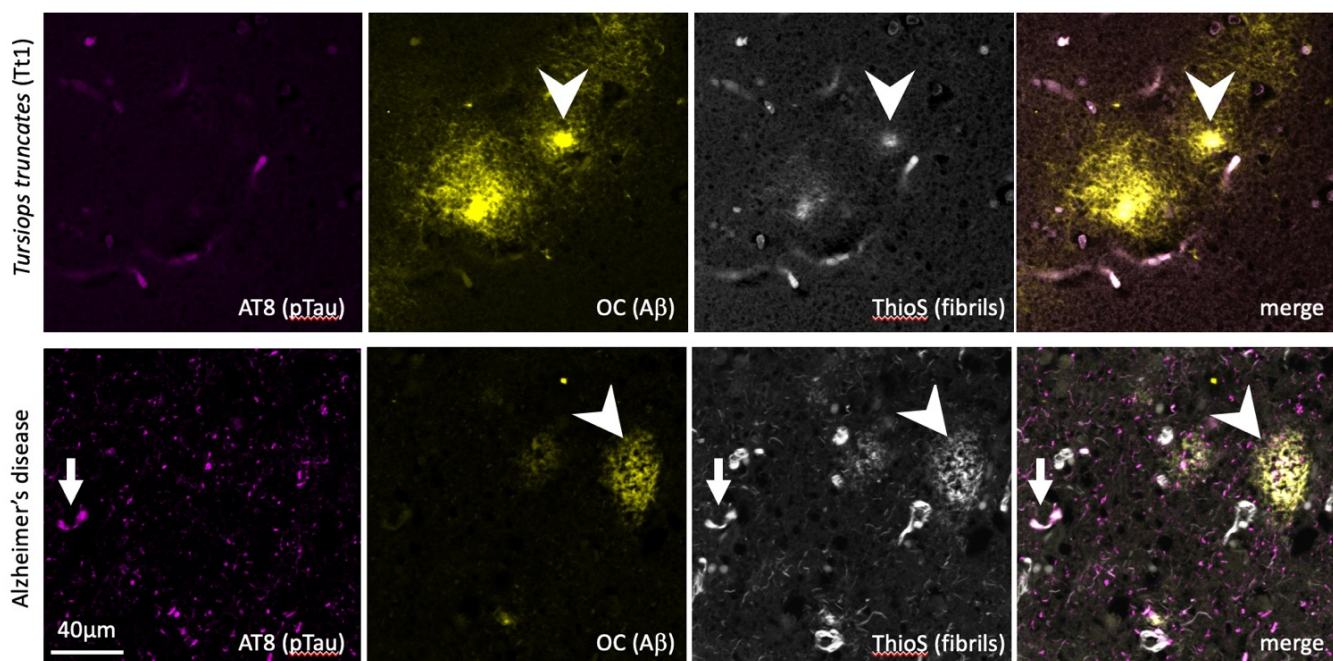

Supplement: Supplementary file 1 — Figure S1. A: Human brain from a patient definitively diagnosed with Alzheimer's disease used as a positive control when labelling for pTau (brown pigment) using antibody AT8. All odontocetes examined failed to label with antibody AT8 despite a positive control section (as above) being included at all times. B: AT8 staining of phospho‐tau (magenta) was not observed in any of the animals in the study (example from animal Tt1 shown top row). Dense cored amyloid plaques labelled with both Aβ antibody OC (yellow) Thioflavin S to stain fibrils (Thioflavin S, grey) were observed (arrowheads). Both AT8 positive Thioflavin S positive fibrillar neurofibrillary tangles (arrows) and dense core plaques were observed in a positive control slide from an Alzheimer's case (bottom). Scale bar 40 μm. [file EJN-57-1161-s001.pdf]
